# AlignRT^®^ and Catalyst™ in whole‐breast radiotherapy with DIBH: Is IGRT still needed?

**DOI:** 10.1002/acm2.12553

**Published:** 2019-03-12

**Authors:** Marko Laaksomaa, Sebastian Sarudis, Maija Rossi, Turkka Lehtonen, Jani Pehkonen, Jenny Remes, Helmi Luukkanen, Tanja Skyttä, Mika Kapanen

**Affiliations:** ^1^ Department of Oncology Tampere University Hospital Tampere Finland; ^2^ Department of Medical Physics Länssjukhuset Ryhov Jönköping Sweden; ^3^ Department of Medical Physics Medical Imaging Center Tampere University Hospital Tampere Finland

**Keywords:** breast cancer, deep inspiration breath hold, image guidance, radiotherapy, surface guidance

## Abstract

**Purpose:**

Surface guided radiotherapy (SGRT) is reported as a feasible setup technique for whole‐breast radiotherapy in deep inspiration breath hold (DIBH), but position errors of bony structures related to deeper parts of the target are not fully known. The aim of this study was to estimate patient setup accuracy and margins obtained with two different SGRT workflows with and without daily kV‐ and/or MV‐based image guidance (IGRT).

**Methods:**

A total of 50 breast cancer patients were treated in DIBH, using SGRT for the patient setup, and IGRT for isocenter corrections. The patients were treated at two different departments, one using AlignRT^®^ (25 patients) and the other using Catalyst™ (25 patients). Inter‐fractional position errors were analyzed retrospectively in orthogonal and tangential setup images, and analyzed with and without IGRT.

**Results:**

In the orthogonal kV‐kV images, the systematic residual errors of the bony structures were ≤ 3 mm in both groups with SGRT‐only. When fine‐adjusted by daily IGRT, the errors decreased to ≤ 2 mm; except for the shoulder joint. The residual errors of the ribs in tangential images were between 1 and 2 mm with both workflows. The heart planning margins were between 3 and 7 mm.

**Conclusions:**

The frequency of IGRT may be considerably reduced with a well‐planned SGRT‐workflow for whole‐breast DIBH with residual errors ≤ 3 mm. This accuracy can be further improved with an IGRT scheme.

## 1 INTRODUCTION

Deep inspiration breath hold (DIBH) generally leads to lower doses to the heart and lungs than irradiation in free breathing (FB) and has become daily practice worldwide in the radiotherapy (RT) of left‐sided breast cancer.^1^, ^2^ The radiation‐induced risk for long‐term perfusion and wall motion abnormalities in the heart is thus smaller with DIBH than in FB treatment.^1^ The breath hold (BH) can be achieved with various techniques such as voluntary BH, optical tracking of the chest wall, and spirometric methods.

Conventional patient setup relies on tattoo marks and lasers. With such setup, isocenter variation in DIBH breast RT is up to 4.4 mm and daily MV‐ or kV‐based image guidance (IGRT) is recommended.^3^, ^4^ However, surface guided radiation therapy (SGRT) has recently commenced to enhance or substitute the tattoo‐based setup, possibly improving the setup accuracy and the reproducibility of the DIBH position.^5^, ^6^, ^7^, ^8^ SGRT monitors the patient′s surface optically and compares it to the planned reference surface.^5^, ^9^ AlignRT^®^ (VisionRT, London, Great Britain) and Sentinel/Catalyst™ (C‐Rad, Uppsala, Sweden) are the current two most used systems for SGRT.

With both AlignRT^®^ and Catalyst™, sub‐millimeter accuracy has been reported in phantom studies.^10^, ^11^ In patients, the accuracy is influenced by several additional factors, such as the fixation method, workflow at the CT, BH training, the absolute BH level (BHL), setup procedures, selection of region of interest (ROI) for the SGRT, tissue deformations such as swelling, protocols for IGRT, and tolerances for the SGRT and IGRT. Intra‐fractional setup variation is small for lymph node negative (N0) breast treatments with tangential field arrangement.^12^, ^13^ However, unsuccessful patient setup is a major contributor to residual errors in DIBH.^3^ The advantage of SGRT over conventional tattoo‐ and laser‐based setup is the large surface for patient positioning compared to a few isolated marks. Furthermore, both the FB and BH surfaces are available for patient positioning. However, the longitudinal accuracy is limited due to flatness of the body surface in this direction. During treatment, the intra‐fractional movement of the patient surface and/or isocenter can be monitored in six‐dimensional (6D) and radiation is allowed only when the patient is within the given thresholds.^14^, ^15^

The heart position in DIBH is reported to have daily variability up to ± 10 mm.^16^ The advantage of DIBH over FB in reducing the dose to the heart depends on BHL reproducibility during treatment.^17^ In breast‐only (N0) RT using DIBH, tangential images can be considered as the most important estimator for the accuracy of the actual treatment delivery if tangential beams are used.^18^ In previous studies, good inter‐fractional accuracy has been achieved in N0 DIBH with margins of 5–6 mm.^4^, ^13^, ^19^ However, it is known that the errors of patient posture and isocenter in lateral (LAT) and anterior–posterior (AP) directions can be underestimated in tangential images.^20^ Patient rotation may increase the heart dose, arm position lower than planned may increase the dose to the shoulder joint,^4^ or the heart dose and irradiated lung volume may increase if BHL is too shallow^17^, ^21^ — all of which are poorly visualized in tangential images.

The purpose of this retrospective study was to evaluate the inter‐fractional setup accuracy of N0 SGRT‐guided DIBH patients with and without IGRT to determine if daily IGRT is still needed with SGRT. The accuracy was studied in terms of isocenter error and residual errors of bony landmarks for the workflows of two RT centers using SGRT. Residual setup errors and required margins for PTV and organs at risk (OAR) with and without IGRT were calculated.

## 2 MATERIALS AND METHODS

### 2.A Patients

**Figure 1:**
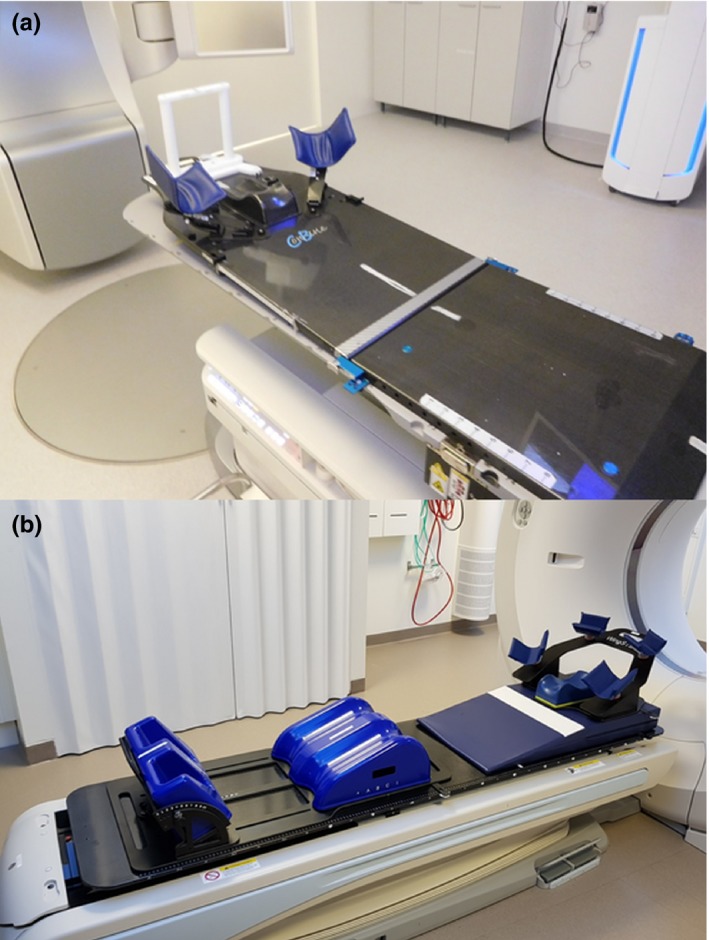
Fixation in Group A (a) and Group C (b).

This study investigated the setup errors for 50 left‐sided breast cancer patients receiving adjuvant whole‐breast RT. The DIBH technique was used for all left‐sided breast patients with age <70 yr or otherwise healthy <75 yr (Group A), or age ≤80 yr (Group C). 25 of the patients were positioned with AlignRT^®^ (Group A = Align) and 25 patients were positioned with Catalyst™ (Group C = Catalyst). The mean patient ages were 57 yr (range 44–76; Group A) and 60 yr (range 40–80; Group C). Candor's ConBine fixation device (Candor, Gislev, Denmark) and WingSTEP™ (Elekta Ltd, Stockholm, Sweden) with a soft wedge and the legs on a Prostep (Elekta Ltd) were used for patient immobilization in Group A and Group C, respectively. The soft wedge was used in Group C to tilt the patient upward toward the Sentinel camera to allow for better sternum view. The immobilizations are presented in Fig. 1.

### 2.B Planning CT

The planning CT was performed with a visually guided DIBH technique at 120 kVp with either a Philips Brilliance Big Bore (Philips Medical Systems, Eindhoven, The Netherlands) (Group A) or Toshiba Aquilion LB (Toshiba Medical System, Tokyo, Japan) scanner (Group A, C). The slice thickness was 3 mm. In both groups the midline tattoo was set on the sternum, and the lateral tattoos dorsally out of the soft breast tissue. Group A used an additional tattoo 20 cm caudally from the mid tattoo to control for patient yaw. Patients in Group A were guided with the RPM™ system (Varian Medical systems, Palo Alto, USA) with a patient monitor for visual DIBH guidance. The patients in Group C were guided with the Sentinel™ (C‐Rad, Uppsala, Sweden) system with wireless goggles. The gating points at the CT were two dot markers on the sternum in Group A and a virtual point on the processus xiphoideus in Group C. The gating window was 3 mm (±1.5 mm) in both groups. In Group C only a DIBH‐CT was acquired, while in Group A an additional FB scan was acquired for 20 of the patients.

The patients were trained on BH technique on the CT‐couch before the DIBH‐imaging. The patients in Group A were advised to inhale as much as comfortably possible and hold their breath for at least 20 s. The patients in Group C were instructed for partial DIBH based on national recommendations.^22^ Thus, the vertical change between FB and DIBH was not larger than 16.5 mm in Group C.^22^ The CT time scheduled for the DIBH patients, including training, was 60 min at both centers.

### 2.C Treatment

Patients were treated to 42.56 Gy in 16 fractions (n = 46) or 50 Gy in 25 fractions (n = 4) with field‐in‐field technique consisting of two or three tangential 6‐MV fields on Varian TrueBeam treatment machines (Group A, C). The PTV was contoured in both units based on ESTRO guidelines.^23^ Daily orthogonal kV and/or tangential MV imaging was used for all patients. The treatment started 10–12 days (Group A) or 3–6 days (Group C) after the CT.

### 2.D SGRT

With both AlignRT^®^ and Catalyst™, the position of the patient was monitored with three cameras. The optical system compared the actual patient surface with the reference surface from the planning CT or from a reference surface acquired optically in the treatment room. Initial positioning was aligned with tattoos and thereafter fine‐tuned with SGRT. DIBH guidance was performed with SGRT.

#### 2.D.1 Group A

**Figure 2:**
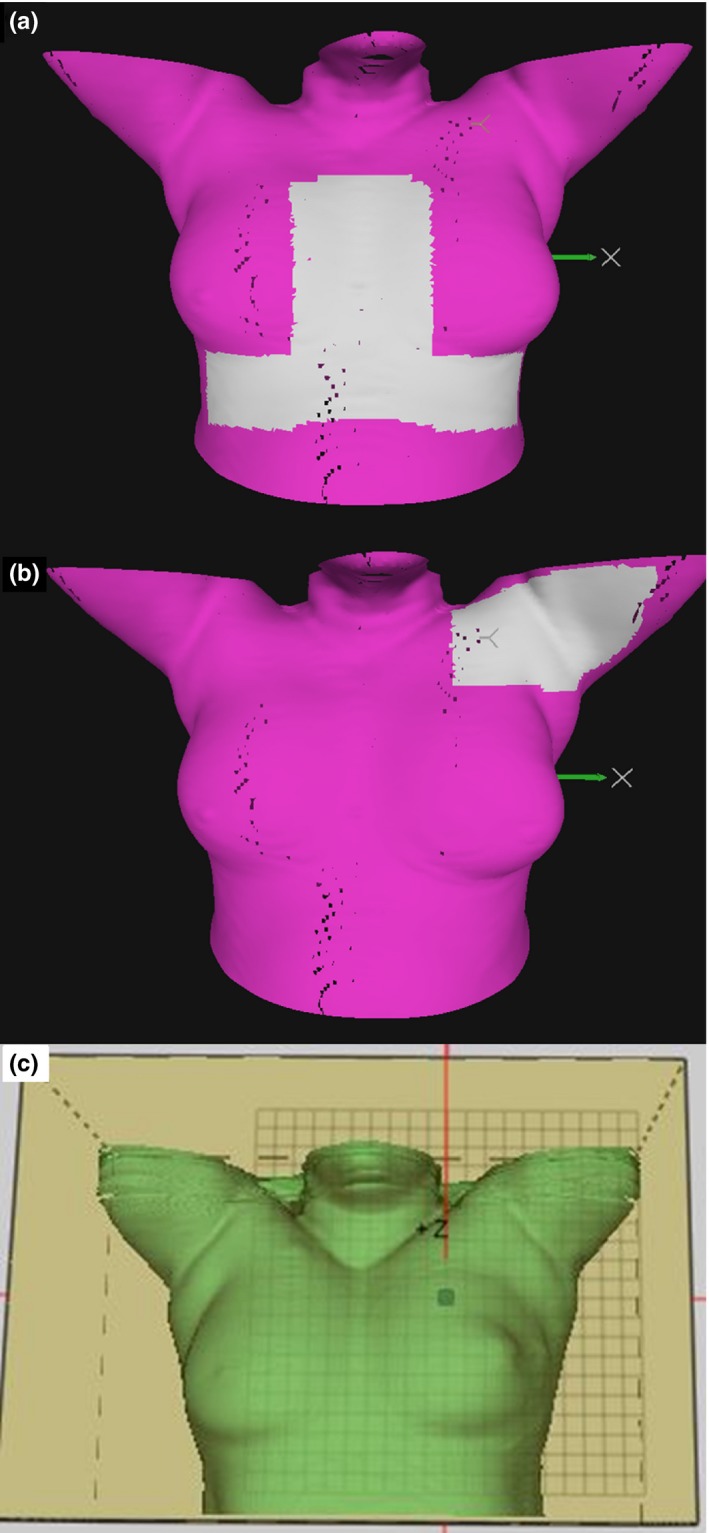
Region of interest (ROI) for surface guidance for Group A as the white shaded area (a). The additional ROI for Group A for possible arm position corrections (b). The ROI of Group C as the large green surface limited by the box (c).

The ROI was determined to include bony structures of the chest wall but to mostly exclude the soft tissue of the breast to diminish the effect of soft tissue swelling or deformation [Fig. 2(a)]. However, the medial part of the soft tissue with curvature shaping was included for the aid of better optical localization of the longitudinal direction. An additional ROI was used for arm positioning [Fig. 2(b)]. In both FB and BH setup 1 mm/degree accuracy was aimed for, and correct (<1 mm) isocenter according to AlignRT^®^ was achieved with manual couch shifts. During treatment, BH surface tolerances for real time deltas (RTD) were set to ± 3 mm for translations, except for the AP direction where it was set to ± 2 mm, resulting in a 4‐mm BHL gating window. The tolerance for the magnitude was set to 4 mm, and for the rotations to ±3 (yaw, roll and pitch). The initial FB surface originated from the CT (n = 20) or from the first treatment (n = 5). The initial FB surface was replaced to achieve better patient position in cases where systematic errors were seen in the IGRT at the first three fractions. For the BH instead, the initial surface was primarily used. New BH surface was recorded only if systematic errors in the isocenter were larger than 5 mm. The vertical couch value was acquired after the first three fractions based on IGRT to standardize the location of the spine.

#### 2.D.2 Group C

A box‐shaped ROI was used which included the whole chest wall (breasts) and the lower parts of the chin and arms [Fig. 2(c)]. In addition to the visual setup data on the screen, a colormap was projected on the patient skin to guide the positioning. No FB surface was available from the CT, but the FB reference surface was acquired at the first fraction after patient alignment based on orthogonal kV‐imaging. From the second fraction, 3D automatic couch shift was performed to shift the patient to the calculated isocenter position based on Catalyst™. In both the FB setup and during the treatment, an 8‐mm surface tolerance was used for the entire ROI [Fig. 2(c)], ±3° tolerance for the pitch, roll and yaw, ±4 mm tolerance for isocenter, and ± 1.5 mm for the BHL (3‐mm gating window).

### 2.E IGRT protocol

**Figure 3:**
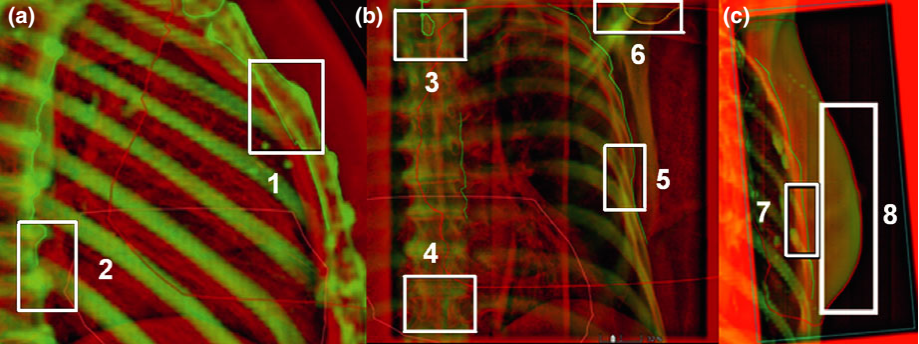
Matching locations mid sternum (1) and vertebrae (2) in the LAT image (a); Th1‐2 (3), Th8‐10 (4), ribs (5), and shoulder joint (6) in the AP image (b), and ribs (7) and soft tissue (8) in the tangential image (c).

The matching points used for patient positioning and evaluation are presented in Fig. 3. The BHL was measured between Th6‐7 and sternum [Fig. 3(a)].

#### 2.E.1 Group A

In the first three fractions, both LAT and AP kV and tangential MV setup images were acquired for all patients in BH. Tangential images only were acquired from the fourth fraction onwards if tolerances were not exceeded on the first three fractions in IGRT verification. However, at least eight additional AP + LAT images were acquired during the treatment for all patients. Thus, at least 11 AP + LAT images were acquired during the treatment course.

In the LAT images, the AP and CC directions were first matched based on vertebrae (Fig. 3). For the sternum, the location was determined using AlignRT^®^ irrespective of the couch VRT. The BHL was thus based on kV‐based vertebrae match and optical‐tracking‐based sternum position, and correct BHL was verified with another LAT image after couch shifts. The couch VRT was acquired after the first three fractions, diminishing the need for two LAT images on the next fractions. LAT was matched on the middle of the ribs in the AP images.

The tolerances were 10 mm for patient yaw rotation (measured in the vertebrae between Th1 and Th10), ±5 mm for isocenter in all directions, and ±3 and ±4 mm for BHL in AP and LNG, respectively. The action limit for the shoulder joint was 8 mm if it was shifted toward PTV and into the field.

#### 2.E.2 Group C

Daily AP + LAT kV setup images were acquired in BH, and the isocenter error was corrected before each fraction. Tangential images were acquired on the first three fractions only. The action limit for the displacement error was ±4 mm in BHL. Action limits for the shoulder joint were not applied. The AP direction was matched on the sternum. The CC direction was matched to a compromise between the sternum and the middle of the ribs, and the LAT direction was matched on the middle of the ribs.

### 2.F Errors in isocenter and bony structures

The residual isocenter errors of SGRT were evaluated as the couch shifts required based on the online match of the orthogonal kV‐kV‐images after the SGRT‐based isocenter positioning. Patient yaw rotation was evaluated from the daily variation between Th1 and Th10 in the AP image. For the accuracy of the BHL, displacement errors between Th6‐7 and the sternum were evaluated in the LAT kV image.^24^ For the arm position, displacement errors between Th1 and shoulder joint were evaluated in the AP kV image.

Errors in the estimated landmarks were evaluated after the couch vertical position was acquired after the first three fractions (Group A) or after the FB surface recording at the first fraction (Group C). The errors were calculated for mid vertebrae, the ribs, the sternum, and for the CC compromise between the ribs and sternum after IGRT corrections. The errors for SGRT‐only were calculated offline by subtracting the SGRT‐based isocenter error from the residual errors of the bony landmarks.

The setup margins for the PTV were calculated using the van Herk's formula (2.5Σ + 0.7σ) where Σ denoted the systematic error and σ the random error, both expressed as one standard deviation.^25^, ^26^ The orthogonal margins were intended for volumetric modulated arc therapy (VMAT), and the tangential skin margins were estimations for the sufficiency of skin flash in VMAT treatments.

### 2.G Errors and margins for OAR

The Stroom and Heijmen's margin recipe (1.6Σ + 0.2σ)^27^ was used for calculating the planning margin for the heart and shoulder joint. The OAR margins for the heart were based on the heart shadow in portal imaging, where AP and LAT were combined. LNG was not evaluated due to uncertainties in image analysis. The OAR margins for the shoulder joint were based on the AP image.

### 2.H Statistical analysis

Statistical tests were used to evaluate the differences between the workflows. The Mann–Whitney *U* test was used to test for equality of means. The two‐tailed *F*‐test was applied to test for equality of variances. A *P* ≤ 0.05 was considered statistically significant.

## 3 RESULTS

### 3.A Errors in isocenter and bony structures

The residual errors of the SGRT‐guided isocenter are presented in Table 1. The calculated PTV margins are presented in Table 2.

**Table 1:** Errors of the isocenter, breath hold level (BHL), patient yaw rotation, and shoulder joint after patient alignment with optical surface monitoring. Image direction anterior–posterior (AP) or lateral (LAT) is in parenthesis for each structure. Patient yaw is shown as the displacement errors between Th1 and Th10. The residual errors in tangential images are reported after isocenter corrections. Errors are presented as systematic error Σ + random error σ in mm

	Group A			Group C		
	AP	CC	LAT	AP	CC	LAT
Isocenter (AP‐LAT)	0.6^a^ + 1.8	1.8 + 2.9	1.1^a^ + 1.9	1.1 + 2.1	1.6 + 2.3	2.0 + 1.7
BHL (LAT)	1.4 + 2.0^b^	2.5 + 3.2^b^		1.7 + 1.4	1.6 + 1.7	
Yaw, (AP)		0.5^a^ + 0.8	2.0 + 2.2^b^		0.3 + 0.6	1.6 + 1.6
Th1‐shoulder joint (AP)		3.7 + 3.8^b^	1.8 + 2.0		3.1 + 2.4	2.0 + 2.1
Ribs (tangential)^c^	1.0 + 1.2	0.6 + 1.1^b^		1.5 + 1.8	0.6 + 0.7	
Soft tissue (tangential)^c^	1.5 + 1.7	1.2^a^ + 1.8		1.5 + 1.6	2.2 + 1.8	
Shoulder (tangential)^c^	1.4^a^ + 1.9	3.3 + 3.5^b^		2.4 + 2.1	2.9 + 2.4	

a Significant difference in systematic errors between the groups (f‐test *P* < 0.05).

b Significant difference in random errors between the groups (Mann‐Whitney *P* < 0.05).

c AP/LAT are combined.

**Table 2:** Planning margins for the PTV and for the organs at risk, presented in mm

	Group A			Group C		
	AP	CC	LAT	AP	CC	LAT
PTV (AP‐LAT)						
SGRT only	3	6	4	4	6	6
SGRT + IGRT	3	5	3	3	4	4
PTV (tangential)^a^						
Soft tissue	5^a^	4		5^a^	7	
Heart (tangential)^a^	7^a^			3^a^		
Shoulder (AP)		6	3		5	4

a AP/LAT are combined.

With SGRT‐based setup, IGRT showed that 10% of the isocenter errors exceeded a 5‐mm limit in the CC direction in the compromise between the ribs and sternum in both groups. The corresponding percentages for the ribs in the LAT direction were 5% and 8%; and for the sternum in the AP direction 6% and 2% in Group A and Group C, respectively. The systematic errors were below 3 mm with SGRT‐only.

The residual errors reduced when switching from SGRT‐only to daily IGRT (Table 3). With daily IGRT, the systematic errors were below 2 mm in both groups, but the random CC‐error of the sternum in Group A was larger than 2 mm (Table 3). For both groups, the differences in the systematic and random errors of the vertebrae were not significant in the AP direction between using SGRT‐only and daily IGRT. In Group A, the random residual error of the sternum did not improve in CC nor AP direction. In Group C, the systematic CC‐error of the sternum did not improve. All other residual errors were significantly lower (*P* < 0.05) with SGRT + IGRT than with SGRT only.

**Table 3:** Residual errors of bony landmarks after patient alignment with optical surface monitoring and after image guidance. Errors are presented as systematic error Σ + random error σ in mm

	Group A			Group C		
	AP	CC	LAT	AP	CC	LAT
SGRT						
Vertebra (LAT)	1.4 + 2.0	2.8 + 3.5		1.6 + 1.6	1.8 + 2.4	
Sternum (LAT)	1.7 + 1.7	2.4 + 3.0		1.4 + 1.4	2.2 + 2.2	
Ribs (AP)		2.6 + 3.3	1.8 + 1.9		2.1 + 2.5	2.2 + 1.9
Sternum and ribs (AP)		2.1 + 2.8			2.3 + 2.1	
SGRT + IGRT						
Vertebra (LAT)	1.0 + 1.4	1.3^a^ + 1.9^b^		1.3 + 1.3	0.8 + 1.1	
Sternum (LAT)	1.1 + 1.7^b^	1.7 + 2.7^b^		0.8 + 1.1	1.8 + 1.9	
Ribs (AP)		1.4^a^ + 2.0	0.9 + 1.2		1.0 + 1.7	0.9 + 1.3
Sternum and ribs (AP)		1.1 + 1.7			1.2 + 1.3	

a Significant difference in systematic errors between the groups (f‐test *P* < 0.05).

b Significant difference in random errors between the groups (Mann‐Whitney *P* < 0.05).

Tangential images were taken as confirmation after AP + LAT images (n = 368, including both Group A and C), or as the only imaging (n = 102, Group A). In the tangential images (Table 1), the systematic residual errors in both AP/LAT and CC directions were less than 2 mm in both groups. In none of the investigated 470 tangential images the 5‐mm limit was exceeded in the CC direction, nor the 4‐mm limit in the AP/LAT direction. In Group A, during the 102 fractions with tangential images with no prior AP/LAT images, in only 1% the 5‐ and 4‐mm tolerances were exceeded.

### 3.B Errors and margins for OAR

The mean amplitude of the BHL was 18.4 mm (range from 10.8 to 34.2 mm) in Group A and 11.5 mm (range from 9.5 to 16.5 mm) in Group C. In portal imaging, the heart distance from the field border changed from the planned 3.4 ± 7.2 mm to actual treatment values of 4.9 ± 8.1 mm in Group A; and from 7.2 ± 4.2 mm to 7.8 ± 4.5 mm in Group C. The OAR margins for the heart and shoulder joint are presented in Table 2.

## 4 DISCUSSION

The setup accuracy in image‐guided radiation therapy (IGRT) is well studied, achieving 4.3–8.4 mm margins in portal images (2D).^3^, ^4^, ^14^, ^17^ However, surface guidance may enhance the reproducibility of patient setup, and potentially decrease the need for image guidance. In this study, the systematic isocenter accuracy was ≤2 mm in all directions with a SGRT + IGRT workflow. The systematic isocenter errors of up to 2 mm in all three directions were acceptable with SGRT only, a clear improvement compared with up to 4 mm using conventional laser setup.^4^ The clinical importance of systematic residual errors reducing from 3 mm with SGRT‐only to 2 mm with the combination of SGRT and IGRT may be questionable; however, also the random errors were reduced with the addition of IGRT.

In previous RPM™ studies using tattoo‐based setup, random isocenter error of up to 4.4 mm has been reported in the CC direction and daily IGRT is needed.^4^ The most used landmarks for AP/LAT matches are the ribs and sternum.^4^ In the current work, SGRT with either workflow led to smaller errors in these landmarks than the tattoo‐based errors. In the AP‐direction, the discrepancy was small between SGRT and IGRT. With SGRT‐only the 5‐mm limit was exceeded in 10% of the fractions in the CC direction. The flat shape of the chest wall with limited curvature in the CC direction is challenging for accurate SGRT localization. Furthermore, the cranial breathing movement may not always reproduce similarly in the soft tissue.^28^ Daily or at least weekly image guidance, based on patient‐specific consideration, is therefore still needed.

Couch corrections in 6D could decrease the errors in yaw, roll, and pitch.^29^ There were some patients in both groups where SGRT detected pitch, but the users were unable to correct the error in the FB setup. In Group C there was more tilt in the fixation device than in Group A. This may force the patient vertebrae towards the couch, thus better reproducing the patient setup. The yaw rotation errors in both groups were comparable with the tattoo‐based setup using additional tattoo for yaw,^4^ as both SGRT and tattoo setups result in 2‐mm systematic and random errors.^4^ As the SGRT setup was prepared with tattoo‐based setup in both Group A and C, it may be concluded that SGRT did not improve yaw compared to the laser setup.

The BHL was reproducible in both groups in the AP direction, but the random AP and CC errors were larger in Group A (BHL ± 2 mm) than in Group C (BHL ± 1.5 mm). The difference (0.5 mm in either direction) was probably too small to outweigh other sources of error. The random errors are more likely to arise from unsuccessful patient setup and inter‐fractional changes in the breathing technique. Also the required heart planning margins of Group A were larger than those of Group C, which is likely due to patient cheating by lifting the back, related to pitch and CC errors. In full DIBH (Group A) the patient might start lifting her back on days with decreased physical capacity, whereas in partial DIBH (Group C) she may still reach the desired BHL. In a previous study by Skyttä et al., the gating window level tracked on sternum was corrected by 2.7 mm on average in 26 patients (range 1.9–7.0 mm).^17^ On average, the mean heart dose decreased by 0.6 Gy, and V(10 Gy) decreased from 4.5% to 2.8% with the correction. Gaining such decrease with only 2.7‐mm correction compared to 6.9‐mm average difference between full and partial DIBH in this study, the larger uncertainties associated with full DIBH might be justified. However, this should be verified with the same patients imaged in full and partial DIBH, and is outside the scope of this study.

IGRT‐based corrections for isocenter position or BHL, especially in the AP direction, were easier to perform with Catalyst™ than with AlignRT^®^ due to differences in the user interface of the current software versions. Taking new reference BH surface after isocenter corrections while simultaneously considering the correct BHL was slightly more complicated with AlignRT^®^ than with Catalyst™. The BHL errors of SGRT‐only in Group A (AP 1.4 ± 2.0 mm, CC 2.5 ± 3.2 mm) were similar to the BHL‐corrected data with the sternum‐based RPM™ tracking and IGRT (AP 2.1 ± 1.6 mm, CC 2.8 ± 2.6 mm),^17^ and in Group C, the errors were even smaller (AP 1.7 ± 1.4 mm, CC 1.6 ± 1.7 mm). Because the systematic residual errors in the BHL were ± 2 mm, clinically significant changes of lung volume were not likely to exist in either group.

For VMAT, PTV margins 3.7–4.9 mm have been previously published with cone beam CT images (3D)^19^ The 3D margins in this study were similar: 3–6 mm with SGRT only, and 3–5 mm with SGRT+IGRT. For soft tissue deformations on the skin contour, the use of 5‐mm skin flash with 8‐mm optimization bolus has been suggested.^30^ In this study, the soft tissue margins of 5 mm in the combined AP‐LAT direction in the tangential images were within the optimization bolus thickness.

In tangential images, Schönecker et al.^31^ found 1.6‐mm deviation in the distance from the field edge to the ribs in portal images. Also in the present study 1–2 mm random deviation was found (Table 1). In the AP/LAT direction the systematic errors of 1.0 and 1.5 mm in Group A and Group C, respectively, were associated with respective BHL errors of 1.4 and 1.7 mm. In the CC direction the BHL errors of 2.5 and 1.6 mm in both groups were associated with only 0.6‐mm errors in the tangential images, indicating that errors in pitch or changes in the breathing technique are poorly visualized in tangential images. Imaging the LAT direction for BHL verification cannot be therefore fully omitted and this may be further emphasized when treating lymph‐node positive breast cancer with higher heart and lung doses.

The risk of shoulder mobility disturbances or pain increases with increasing radiation doses to the shoulder joint.^32^ The systematic position error was at the same level in both groups leading to OAR margins of up to 6 mm. In Group A, an additional arm‐ROI was manually changed on the monitor during patient setup because simultaneous following of two ROIs was not possible with AlignRT^®^. The overall position of the patient may therefore change during arm position correction making the workflow unpractical. Larger thresholds were therefore allowed for the arm. Additionally, the CT structures in the DICOM reference did not cover the arms enough in the CC direction. In Group C, the systematic CC error may derive from the FB reference of the first treatment day. If the arm position is incorrect on the first fraction, this error guides the arm positioning throughout the treatment course. The 2.4‐mm random error in the CC direction suggests good reproducibility of the arm with Catalyst™ if the systematic error is corrected.

## 5 CONCLUSION

After imaging at the first fractions, Surface Guided Radiation Therapy (SGRT) without IGRT is feasible for DIBH treatments of the breast with systematic isocenter accuracy within 3 mm. If the SGRT workflow is combined with daily imaging the accuracy can be decreased to 2 mm, and random errors can be reduced. The BHL was accurate within 2 mm, however heart planning margin of up to 3–7 mm may be needed due to errors in pitch and CC movement.

## CONFLICT OF INTEREST

The authors declare none.
